# InterMineR: an R package for InterMine databases

**DOI:** 10.1093/bioinformatics/btz039

**Published:** 2019-01-22

**Authors:** Konstantinos A Kyritsis, Bing Wang, Julie Sullivan, Rachel Lyne, Gos Micklem

**Affiliations:** 1 Laboratory of Pharmacology, School of Pharmacy, Aristotle University of Thessaloniki, Thessaloniki GR-54124, Greece; 2 Department of Genetics, University of Cambridge, Cambridge, UK; 3 Cambridge Systems Biology Centre, Cambridge, UK

## Abstract

**Summary:**

InterMineR is a package designed to provide a flexible interface between the R programming environment and biological databases built using the InterMine platform. The package offers access to the flexible query builder and the library of term enrichment tools of the InterMine framework, as well as interoperability with other Bioconductor packages. This facilitates automation of data retrieval tasks as well as downstream analysis with existing statistical tools in the R environment.

**Availability and implementation:**

InterMineR is free and open source, released under the LGPL licence and available from the Bioconductor project and Github (https://bioconductor.org/packages/release/bioc/html/InterMineR.html, https://github.com/intermine/interMineR).

**Supplementary information:**

Supplementary data are available at *Bioinformatics* online.

## 1 Introduction

Nowadays, the problem of storing, accessing and analyzing huge amounts of data is acutely felt in the life sciences. InterMine constitutes a data warehouse framework, which provides the ability to access, retrieve and analyze rapidly a variety of biological data (Smith *et al.*, 2012). With intuitive tools, like gene set statistical analysis, customized queries and pre-defined templates which incorporate popular queries for specific types of biological data, InterMine databases facilitate the analysis of heterogeneous biological information. Many model organism groups have adopted InterMine (see http://registry.intermine.org) resulting in its use in many studies.

The R programming language is primarily characterized by its powerful statistical and graphical capabilities and is one of the tools of choice for the field of data science (R Core Team, 2008). The language has gained further popularity through its use by Bioconductor, an open source software project based on R, which aims to facilitate the integrative analysis of biological data (Gentleman *et al.*, 2004; Huber *et al.*, 2015). The InterMineR package has been developed to provide access to InterMine databases through the R programming environment, and its formats are compatible with many Bioconductor workflows.

## 2 Implementation

### 2.1 Performing complex queries

InterMineR performs standard HTTP requests to the InterMine web service API through the use of the httr package. The input lists of data identifiers are uploaded to InterMine and the query results returned in the form of JSON or XML before being converted to human readable data.frame or list R objects, which can be easily used for further downstream analysis.

This package provides access to the pre-defined search forms (template queries) of each InterMine instance, which can be used and explored through the getTemplate() and getTemplateQuery() functions, as well as the ability to create user-defined custom queries. Users can assign several different data identifiers as input to these queries and edit or add additional constraints as required. The creation of custom queries in InterMineR is based on the data model of InterMine. Users can define which data they want to select, and constraints can be added to any attribute type, for instance numeric (e.g. genomic locations) and text (e.g. gene identifiers) data types. This enables the users to add, remove or modify existing constraints and set specific constraints on the data that are to be returned from the query. For this purpose, the getModel() function was designed to retrieve detailed information about the available attributes of each InterMine database.

The functions setConstraints() and setQuery() were designed to assist the users in creating custom queries and assigning multiple data identifiers to a specific filter constraint. These functions bypass the manual design and manipulation of lengthy query list objects. Instead both the constraints and the query itself can be defined in two steps, leading to the creation of an R object of the class *InterMineR* which constitutes the final query.

### 2.2 Enrichment analysis

InterMineR also provides an interface between the statistical features of the R language and the gene set enrichment analysis provided by the InterMine framework. Specifically, InterMine provides Gene Ontology enrichment statistics as well as enrichment statistics for other annotation types (Smith *et al.*, 2012). The function getWidgets() can be used to obtain the enrichment analysis ‘widgets’ of InterMine, which can then be used to calculate enrichment for a pre-defined list of biological entities. The hypergeometric distribution is used to calculate significant *P*-values and various methods are available for multiple test correction. To facilitate the visualization of the enrichment analysis results, the function convertToGeneAnswers() was designed. GeneAnswers is an R package that provides statistical and network visualization functions to explore possible relationships between a group of genes and a list of categories (e.g. Gene Ontology terms) (Feng *et al.*, 2010, 2012; Huang *et al.*, 2014) (Supplementary Fig. S1).

### 2.3 Conversion functions for InterMineR query results

For better integration of the InterMineR package in Bioconductor workflows we created two new functions.

convertToGRanges() function converts genomic location data, retrieved by InterMineR queries, to GRanges objects, which constitute scalable data structures for annotated genomic ranges (Lawrence *et al.*, 2013) (Supplementary Table S1). The GRanges package allows a host of range-based operations such as overlap queries and nearest neighbour.

The function convertToRangedSummarizedExperiment() was designed to facilitate the analysis of gene expression data and associated annotations that are retrieved from InterMineR queries. This function converts InterMineR query results to R objects of the class RangedSummarizedExperiment, a flexible class that converts the information about genes (rows), samples (columns) and gene expression values into separate R objects (Morgan *et al.*, 2017).

## 3 Conclusion

Programmatic access to the InterMine data model allows for iteration and repeated performance of complex queries with the option to adjust specific filter constraints and values.

With the InterMineR package complex queries from different InterMine databases can be generated and the results analyzed with the wealth of statistical and graphical tools offered by the R language and the many Bioconductor packages. To facilitate InterMineR usage, vignettes with detailed examples are available in both the Bioconductor project and GitHub repository of the package.

In the future, a graphical user interface will be developed for InterMineR, based on the Shiny framework (Chang *et al.*, 2017). This aims to further simplify the design of custom queries and facilitate the use of the package by novice R users.

## Supplementary Material

btz039_Supplementary_Data
