# Supplementary material for: InterMineR: an R package for InterMine databases
Source: Bioinformatics. 2019 Jan 22;35(17):3206–7. doi: 10.1093/bioinformatics/btz039 (PMC6736411; doi:10.1093/bioinformatics/btz039)
Supplement: btz039_Supplementary_Data [file btz039_supplementary_data.docx]

Supplementary figure 1:

The convertToGeneAnswers() function converts InterMineR enrichment analysis results to an R object of the class *GeneAnswers* in a single step, thus facilitating the interpretation of the enrichment analysis through visualization. To demonstrate the potential of InterMineR in facilitating gene set enrichment analysis and interpretation, GO enrichment analysis was performed on 68 genes associated with diabetes, which can be found in the PL_DiabetesGenes list of the HumanMine database. This is a public gene list and includes genes associated with all forms of Diabetes according to OMIM ([www.omim.org](http://www.omim.org/)). Conversion of the enrichment analysis results to *GeneAnswers* objects allowed for network creation and visualization with GeneAnswers package functions, so showing association between the five most significantly enriched GO terms (Fig. 1A-C). Moreover, five genes (*ABCC8*, *ACE*, *AKT2*, *AQP2*, and *APPL1*), which belong both to the initial gene set and the GO-term-enriched set, were selected for further exploration to identify novel target genes that interact with one or more of them. For the purpose of illustration the five genes were selected at random from the PL_DiabetesGenes list. Only the top five statistically significant GO terms (Hypergeometric distribution, P < 0.05) were selected for display purposes. (A) Barplot displaying enriched GO terms and their respective Benjamini-Hochberg corrected P-values; (B) Network of connections between enriched GO terms and their respective genes. The size of the nodes is proportional to the number of genes in these GO categories; (C) Network of relations between enriched GO terms. The top eight enriched GO terms are shown as large red nodes. Relationships between these GO terms are shown as purple edges. Additional related GO terms are shown as smaller, light red nodes. The relationship between these and the original GO terms is represented with yellow edges. The size of the nodes is proportional to the number genes from the list directly annotated with this term; (D) Network of protein-protein interactions (PPIs) between selected genes (*ABCC8*, *ACE*, *AKT2*, *AQP2*, and *APPL1*) that belong to the enriched GO terms. The five given genes are represented as large black nodes with a yellow frame. Other genes from the original gene list are represented as small black nodes. Small white nodes are genes that are not in the original list, but interact with these genes. Interactions involving one of the five given genes are represented by dark-blue-purple edges. Other interactions are shown with yellow edges. PPI information were retrieved from NCBI using the buildnet() function of the GeneAnswers package.


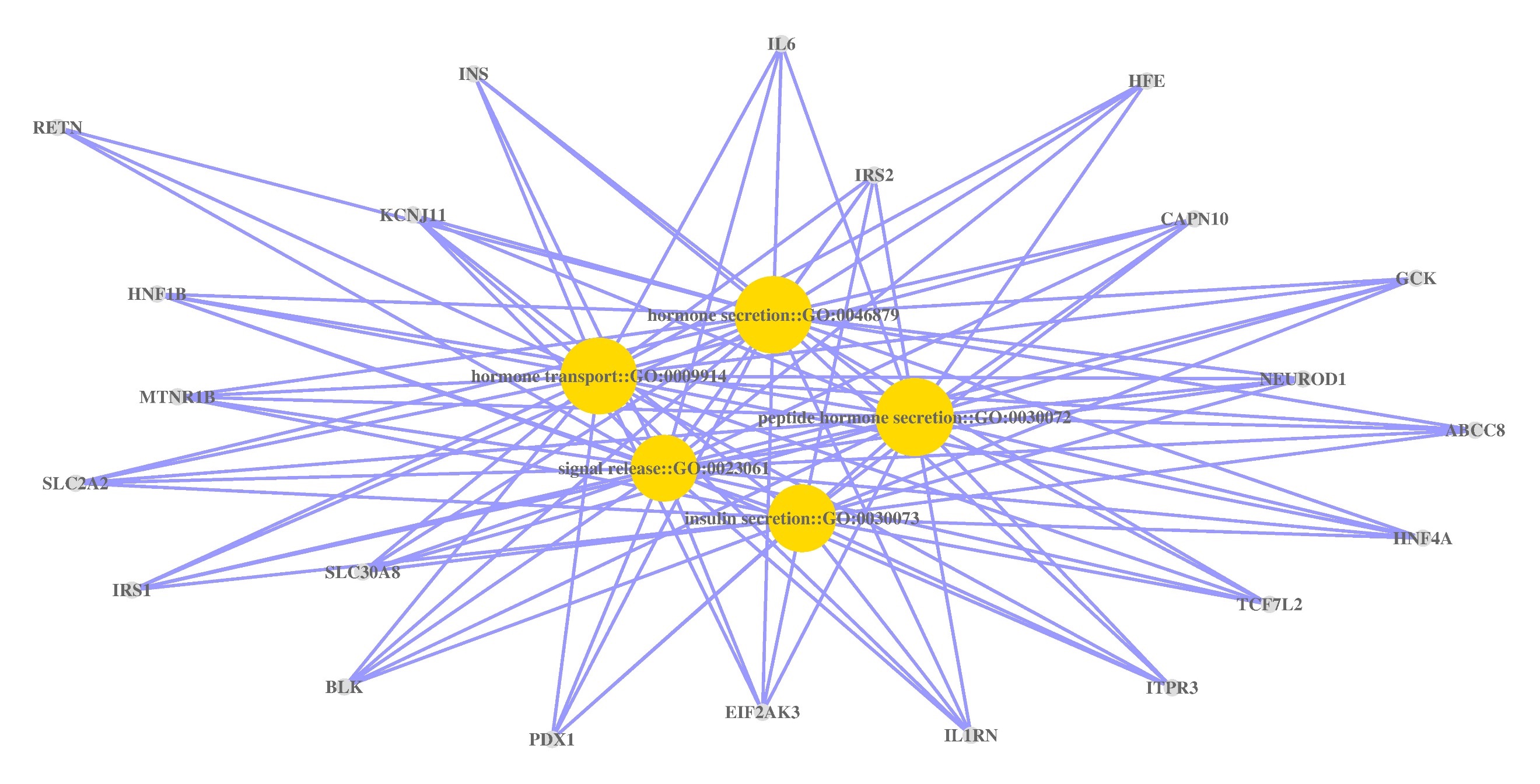

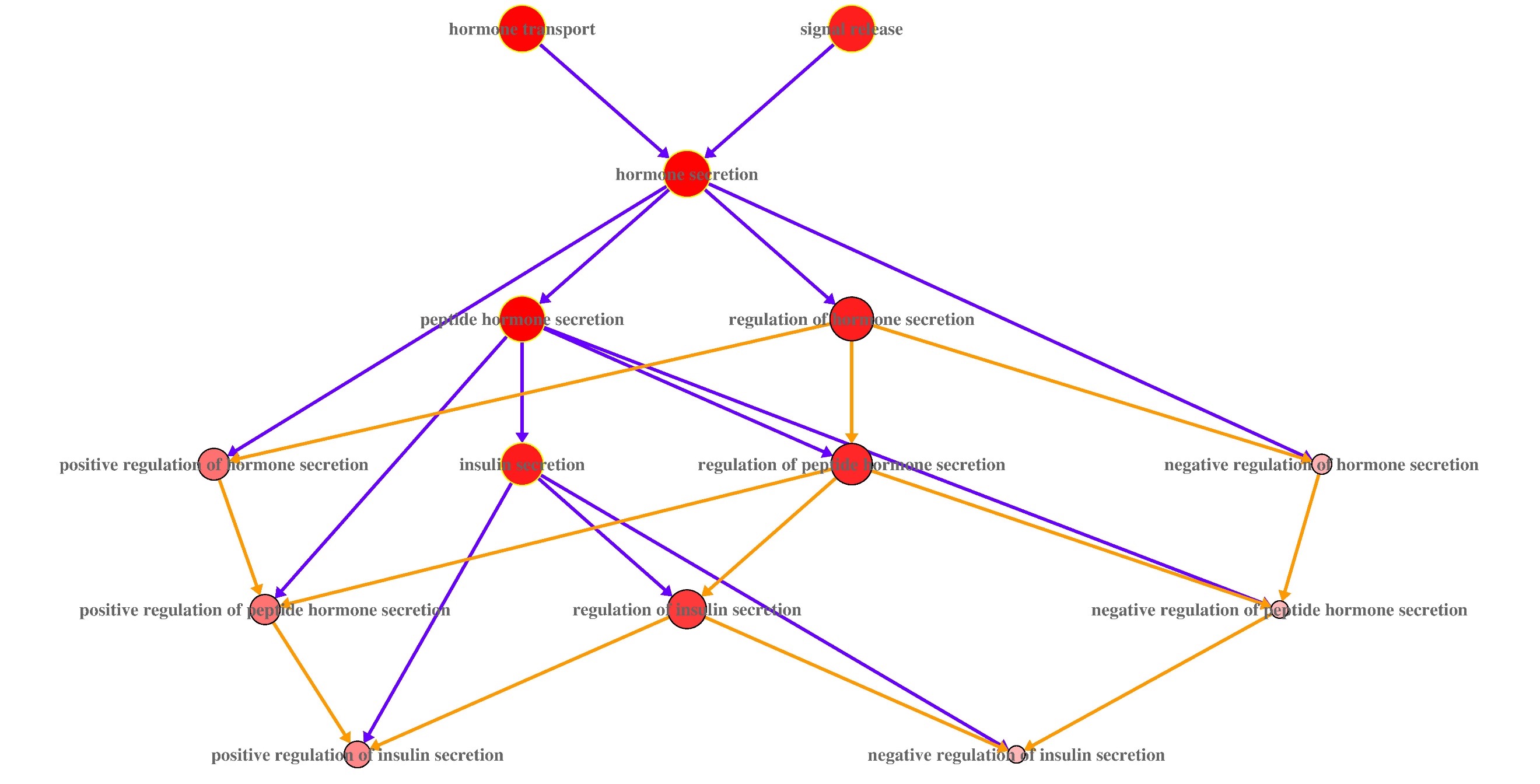


**B**

**A**

**D**

**C**


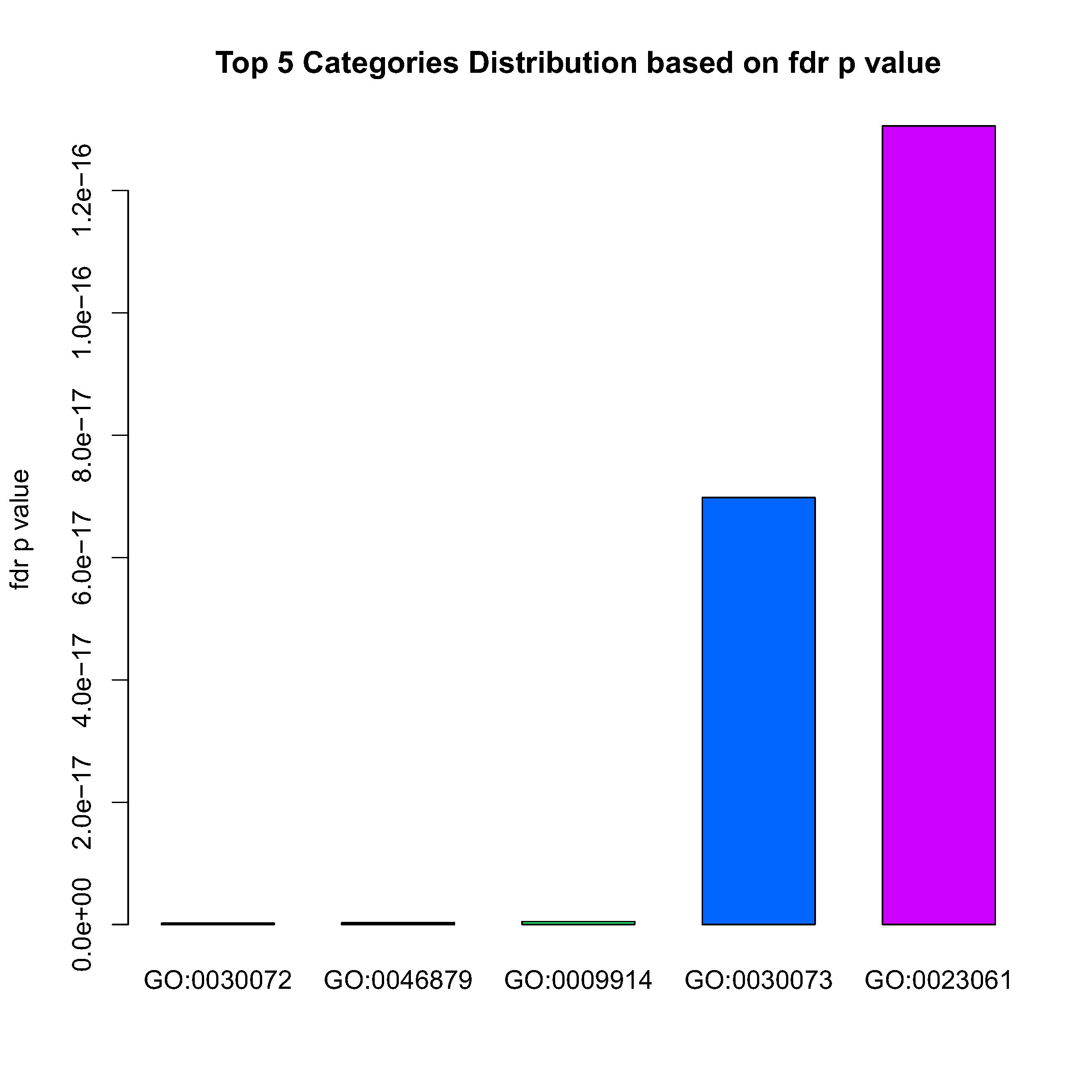

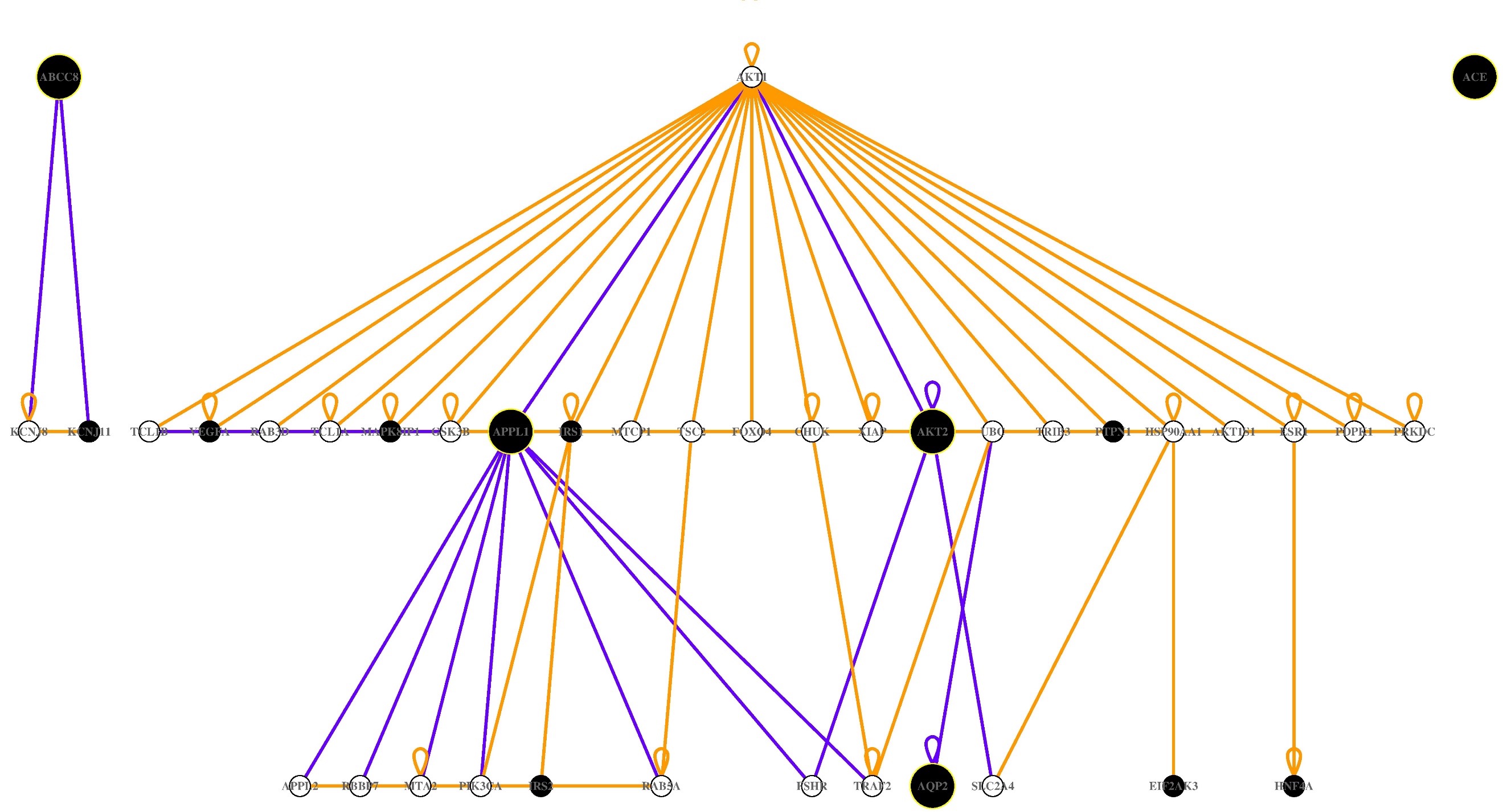


Supplementary table1

The convertToGRanges function. To illustrate this function, the template query ‘Gene_upstreamRegulatoryRegions’ of the FlyMine database was used to retrieve the regulatory elements that are located in the intergenic region upstream of the Drosophila melanogaster gene, *Zen*. The resulting data were converted to GRanges, with a subset shown in the table. This conversion facilitates the application of GRanges operations on the genomic coordinates retrieved by InterMineR and the manipulation of annotations as metadata.

|  | **seqnames** | **ranges** | **strand** | **\|** | **UpstreamIntergenicRegion** | **overlappingFeatures** |
| --- | --- | --- | --- | --- | --- | --- |
| zen | chr3R | [6754487, 6754869] | - | \| | intergenic_region_chr3R_6754195..6755841 | FBsf0000161188 |
| zen | chr3R | [6755181, 6755805] | - | \| | intergenic_region_chr3R_6754195..6755841 | FBsf0000161213 |
| zen | chr3R | [6754261, 6754273] | 0 | \| | intergenic_region_chr3R_6754195..6755841 | TF000823 |
| zen | chr3R | [6754323, 6754350] | 0 | \| | intergenic_region_chr3R_6754195..6755841 | TF000824 |
| zen | chr3R | [6754837, 6755935] | 0 | \| | intergenic_region_chr3R_6754195..6755841 | Unspecified_VT37509 |
| zen | chr3R | [6754137, 6754240] | 0 | \| | intergenic_region_chr3R_6754195..6755841 | zen_0.04 |
| zen | chr3R | [6755182, 6755805] | 0 | \| | intergenic_region_chr3R_6754195..6755841 | zen_dorsal_ectoderm |
